# LMP1 Increases Expression of NADPH Oxidase (NOX) and Its Regulatory Subunit p22 in NP69 Nasopharyngeal Cells and Makes Them Sensitive to a Treatment by a NOX Inhibitor

**DOI:** 10.1371/journal.pone.0134896

**Published:** 2015-08-05

**Authors:** Jian Sun, Chongyu Hu, Yinghui Zhu, Rui Sun, Yujing Fang, Yuhua Fan, Fei Xu

**Affiliations:** 1 Sun Yat-sen University Cancer Center, State Key Laboratory of Oncology in South China, Collaborative Innovation Center for Cancer Medicine, Guangzhou 510060, P.R.China; 2 Hunan Provincial People’s Hospital, No.61 West Liberation Road, Changsha 410005, P.R.China; 3 The First Affiliated Hospital, Sun Yat-sen University, Guangzhou 510080, P.R.China; Gustave Roussy, FRANCE

## Abstract

Oxidative stress is thought to contribute to cancer development. Epstein–Barr virus (EBV) and its encoded oncoprotein, latent membrane protein 1 (LMP1), are closely associated with the transformation of nasopharyngeal carcinoma (NPC) and Burkitt’s lymphoma (BL). In this study, we used LMP1-transformed NP cells and EBV-related malignant cell lines to assess the effects of LMP1 on reactive oxygen species (ROS) accumulation and glycolytic activity. Using NPC tissue samples and a tissue array to address clinical implications, we report that LMP1 activates NAD(P)H oxidases to generate excessive amount of ROS in EBV-related malignant diseases. By evaluating NAD(P)H oxidase (NOX) subunit expression, we found that the expression of the NAD(P)H oxidase regulatory subunit p22^phox^ was significantly upregulated upon LMP1-induced transformation. Furthermore, this upregulation was mediated by the c-Jun N-terminal kinase (JNK) pathway. In addition, LMP1 markedly stimulated anaerobic glycolytic activity through the PI3K/Akt pathway. Additionally, in both NPC cells and tissue samples, p22^phox^ expression correlated with LMP1 expression. The NAD(P)H oxidase inhibitor diphenyleneiodonium (DPI) also exerted a marked cytotoxic effect in LMP1-transformed and malignant cells, providing a novel strategy for anticancer therapy.

## Introduction

Reactive oxygen species (ROS) are byproducts of oxygen metabolism and play an important role in cell signaling and homeostasis. Epstein-Barr virus (EBV), a ubiquitous human herpes virus, is associated with the development of both lymphoid and epithelial tumors. EBV-positive Burkitt’s lymphoma (BL) cells exhibit higher ROS levels compared with EBV-negative BL cells. Additionally, latent membrane protein 1 (LMP1), an EBV-encoded oncoprotein, is hypothesized be a major inducer of ROS [1,2]. LMP1 is a functional homologue of CD40 and a member of the tumor necrosis factor (TNF) receptor family. *Ha YJ* and *Lee JR* demonstrated that CD40 activation produces ROS by activating the NAD(P)H oxidase (NOX) regulatory subunit p40^phox^ vis TNF receptor-associated factor 3 and the phosphoinositide-3-kinase (PI3K) pathways [3]. These studies suggest that NOX might play a role in LMP1-induced ROS induction in human malignancies, However, the detailed molecular mechanism underlying this hypothesis has not been clearly elucidated.

The NOX family is an important intrinsic source of ROS generation. Based on enzyme activity, NOX family members are divided into two groups: catalytic enzymes (NOX1-5 and DUOX 1–2) and regulatory subunits (p22^phox^, p40^phox^, p47^phox^, p67^phox^, Rac1 and Rac2) [4]. The overexpression of NOX subunits often correlates with the development of various types of tumors. For example, human prostate cancers frequently show increased NOX1 [5] and NOX5 [6] levels, and NOX4 plays a critical role in hypoxia-promoted glioblastoma progression [7].

In this study, we aimed to investigate the role of LMP1 in ROS induction in the context of nasopharyngeal carcinoma and to assess the effectiveness of the NOX inhibitor DPI to induce cytotoxicity in transformed nasopharyngeal epithelial cells and cancer cells. We found that LMP1 could enhance p22^phox^ expression in nasopharyngeal epithelial cells. In addition, p22^phox^ was found to be overexpressed in NPC cells, including in malignant cells lacking LMP1 expression, which suggests that p22^phox^ could be an effective target for the NOX inhibitor diphenyleneiodonium (DPI). Furthermore, the glycolytic rate was elevated in LMP1-transformed nasopharyngeal cells, and DPI treatment tremendously increase lactate concentrations. These findings suggest that coupling a high level of aerobic glycolysis with increased LMP1 expression renders the cells vulnerable to DPI.

## Materials and Methods

### Cells line

NP69 cells (NP69 cells harboring *SV40T*) and NP69-LMP1 cells (NP69 cells transfected with pLNSX-LMP1 and stably expressing LMP1) were a kind gift of Dr. George Sai Wah Tsao (University of Hong Kong) (Lo et al. 2003; Tsao et al. 2002). Cells were maintained in serum-free keratinocyte medium supplemented with human recombinant epidermal growth factor (0.1–0.2 ng/mL) and bovine pituitary extract (20–30 μg/mL) (Gibco/Invitrogen Corporation, Carlsbad, California). Cells were incubated at 37°C in a humidified atmosphere with 5% CO_2_.

### Chemicals and Reagents

DPI and 3-bromopyruvate were purchased from Sigma-Aldrich (St. Louis, MO). CM-H2DCF-DA, DAF-FM, HEt, and 2-NBDG were purchased from Invitrogen/Molecular Probes (Carlsbad, CA). SP600125 was acquired from EMD Biosciences (Calbiochem, San Diego, CA). DPI was dissolved in dimethyl sulfoxide (DMSO) and freshly diluted in culture media before used. The final DMSO concentration was less than 0.1% (v/v). In addition, 3-Bromopyruvate was dissolved in water and neutralized with NaOH immediately before use in cell culture. The rabbit polyclonal anti-p22^phox^ antibody was purchased from Santa Cruz Biotechnology (Santa Cruz, CA). Rabbit polyclonal anti-Akt and anti-phospho-Akt (Ser473) antibodies as well as rabbit monoclonal anti-c-Jun and anti-phospho-c-Jun (Ser63) antibodies were purchased from Cell Signaling Technology, Inc. (Beverly, MA).

### Determination of cellular ROS level

Cellular ROS contents were measured by incubating control or drug-treated NP69 and NP69-LMP1 cells with 1 μM CM-H2DCF-DA for 60 min. The cells were then subject to flow cytometry analysis using a FACSCalibur equipped with CellQuest Pro software. For SUNE-1 cells, 4 μM CM-H2DCF-DA was used in a 60-min labeling reaction to obtain sufficient fluorescence signal. CM-H2DCF-DA is a fluorescent probe with a relative specificity for hydrogen peroxide. The peroxide (O_2_-) concentration was measured by flow cytometry in the presence of HEt (100 ng/mL) [8].

### NOX activity assay

DPI is a widely used inhibitor of flavoenzymes, particularly NADPH oxidase. To determine cellular NOX activity, NP69 and NP69-LMP1 cells were lysed in hypotonic phosphate buffer containing protease inhibitors, disrupted by sonication, and centrifuged for 10 min at 1500 rpm. The supernatant, which contained the cytosol and the mitochondrial fraction, was further ultracentrifuged at 100,000 g for 30 min at 4°C. The resulting pellet, which contained the cytosolic membranes and mitochondrial fraction, was resuspended in buffer B (50 mM Tris [pH 7.5], 150 mM NaCl, 1 mM EDTA, protease inhibitor cocktail [one tablet for 10 ml buffer]) and used for the assay. Samples were adjusted to the same protein concentration (1 μg/μL), and 5-μL aliquots were incubated with 94 μL of phosphate buffer (50 mM K_2_HPO_4_, 1 mM EGTA, 150 mM sucrose) and 1 μL of 3-NADPH (4 mM) for 15 min. Before reading, 2.5 μL of 2 mM lucigenin was added. The lucigenin-derived chemiluminescence of the cell homogenates was assessed over 1 min in a 20/20^n^ Tube Luminometer (Turner Biosystem, Sunnyvale, CA).

### Reverse transcriptase-polymerase chain reaction (RT-PCR) for the NAD(P)H subunits and LMP1

Total RNA was isolated from cells using an RNeasy mini kit (QIAGEN, Valencia, CA) according to the manufacturer’s instructions, and cDNA was synthesized as described by Mei et al.^4^ A total of 35 PCR cycles were performed. The primer sequences used for cDNA analysis were presented in S1 Table.

### Assays for cytotoxicity

Cell death was determined by flow cytometry after staining with annexin-V-FITC and propidium iodide (PI) using an assay kit from BD Pharmingen (San Diego, CA) as described.

### Detection of the cellular glycolysis level

The cellular glycolytic activity was evaluated by measuring the glycolytic index as calculated using the following formula: glycolytic index = (L × G) / O, where L is the cellular lactate generation rate, G is the glucose uptake rate, and O is the oxygen consumption rate [9]. To determine the cellular lactate production rate, cells in the exponential growth phase were incubated with fresh medium for 24 h. Aliquots of culture medium were collected for lactate content analysis using an Accutrend lactate analyzer. In addition, a assortment of lactate concentrations in the linear range were used as standards as recommended by the manufacturer (Roche, Mannheim, Germany). Cellular glucose uptake was measured by incubating cells at 37°C in glucose-free RPMI 1640 medium supplemented with 2-NBDG (2-[N-(7-nitrobenz-2-oxa-1,3-diazol-4-yl)amino]-2-deoxy-D-glucose), a fluorescent deoxyglucose derivative used to measure glucose uptake in living cells [10]. After 60 min, 2-NBDG uptake was stopped by the addition of ice-cold phosphate-buffered saline (PBS). The proportion of 2-NBDG-positive cells was detected by flow cytometry. To determine cellular oxygen consumption, cells were resuspended in 1 mL of fresh, warm medium pre-equilibrated with 21% oxygen and placed in a sealed respiration chamber equipped with a thermostat control, a microstirring device, and a Clark-type oxygen electrode disc (Oxytherm, Hansatech Instrument, Cambridge, United Kingdom). The oxygen content in the cell suspension medium was constantly monitored, and the oxygen consumption rate was determined as described previously [2].

### Transit LMP1 plasmid transfection in NP69 cells. The day before transfection

NP69 cells were seeded in six-well plates and grown in antibiotic-free growth medium. The cells were transfected when they achieved 80% confluence. The medium was replaced by serum-free Opti-MEM medium (Invitrogen). Cells were transfected with either pZIP-SV(X) or Pzip-SV(X)-LMP1 using Lipofectamine 2000 (Invitrogen). After incubating the cells for 6 h, Opti-MEM medium was replaced by fresh antibiotic-free growth medium. For subsequent analyses of gene expression and ROS levels, cells were harvested between 24 and 72 h after transfection.

### Real-Time PCR assay

30 NPC and 19 non-cancer nasopharyngeal tissues were collected, and total RNA was isolated using Trizol (Invitrogen). Genomic DNA contamination in RNA preparation was removed using a DNA-free kit (Ambion Inc. Austin, TX, USA). RNA purity was confirmed by the absence of detectable genomic DNA in parallel control PCR reactions using a cDNA sample generated without including the reverse transcriptase (RTase) in the reaction. Total RNA (0.5 μg) was reverse transcribed into cDNA using the Transcriptor High Fidelity cDNA Synthesis kit (Roche) according to the manufacturer’s instruction. Primers for all the tested genes were designed via the Real-Time PCR Primer Design software (GenScript); the primer sequences are listed as below: LMP1 (forward, 5’-CCAAATTTGACGGAAGAGGT-3’; reverse, 5’-AAAGCAGCGTAGGAAGGTGT-3’; cDNA length, 100 bp) and p22^phox^ (forward, 5’-TTCACCCAGTGGTACTTTGG-3’; reverse, 5’-GACGGCCCGAACATCGTAAT-3’; cDNA length, 180 bp). The quantification of p22^phox^ and LMP1 mRNA expression in NPC and non-cancerous tissue samples was performed by cDNA amplification using SYBER Green. The absolute threshold cycle values (Ct values) of the tested mRNAs (GAPDH, p22^phox^ and LMP1) were determined using SDS software v. 2.1 (Applied Biosystems). mRNA expression levels were measured by normalizing CT values of the tested gene with that of GAPDH using the delta Ct method (delta CT = CT_p22phox/LMP1_ –CT_GAPDH_). Then, the correlation of the expression levels of these two genes between NPC and non-cancerous tissue samples was analyzed by Pearson’s correlation method using SPSS16.0 software.

### NPC tissue array Immunohistochemistry (IHC) analysis

The NPC tissue array was purchased from Pantomics (Richmond, CA). The accompanying follow-up data and clinical details provided by this company are available online. IHC was performed as previously described [11]. Images of the sections were independently acquired and quantified by two skilled pathologists. Protein expression was semiquantitatively assessed according to staining intensity; expression was categorized as negative, weakly positive or strongly positive according to a previously described method [11]. Briefly, we employed the scoring guidelines described by Carcangiu et al. Negative samples exhibited an absence of staining, positive membrane staining in less than 10% of the tumor cells, minimal membrane staining in greater than 10% of tumor cells, or positive cells with partially stained membranes. A weakly positive label indicates a weak-to-moderate but complete membrane staining observed in greater than 10% of tumor cells. Strongly positive samples exhibited strong and complete membrane staining in greater than 10% of tumor cells.

### Statistical analysis

All analyses comparing differences between measurements were performed using a one-way analysis of variance (ANOVO) test with SPSS 16.0 software.

## Results

### EBV-encoded LMP1 leads to ROS accumulation in nasopharyngeal epithelial cells

**Fig 1:**
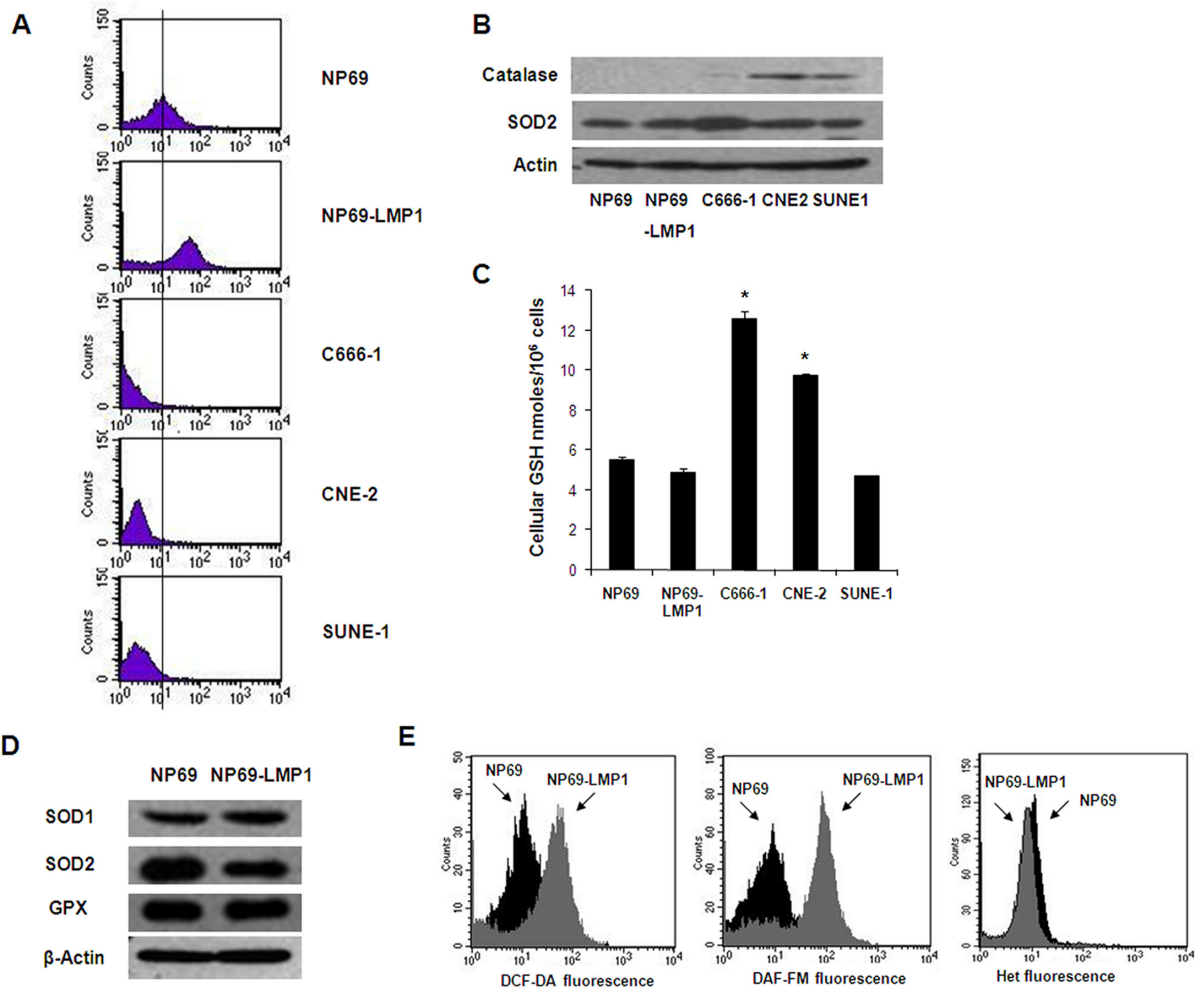
Oncogenic transformation by LMP1 causes increased ROS generation. **A:** Basal hydrogen peroxide levels in immortalized nasopharyngeal epithelial cells (NP69 and NP69-LMP1) and NPC cells (C666-1, CNE-2 and SUNE-1) were detected by flow cytometry using DCF-DA. Each histogram is representative of three experiments. Compared to NP69 cells, NP69-LMP1 cells exhibit significantly higher level of hydrogen peroxide (p<0.05). **B:** Basal protein expression of catalase and superoxide dismutase 2 (SOD2) in immortalized nasopharyngeal epithelial cells (NP69 and NP69-LMP1) and NPC cells (C666-1, CNE-2 and SUNE-1). β-Actin served as a loading control. **C:** Comparison of total cellular GSH in immortalized nasopharyngeal epithelial cells (NP69 and NP69-LMP1) and NPC cells (C666-1, CNE-2 and SUNE-1) (mean ± SD of three experiments, * p<0.05). **D:** Basal protein expression of catalase, glutathione peroxidase (GPX) and superoxide dismutase (SOD1 and SOD2) in NP69 and NP69-LMP1 cells. β-Actin served as a loading control. **E:** Increase in ROS levels in NP69-LMP1 cells detected by flow cytometry using DCF-DA (left panel) and DAF-FM (middle panel). Superoxide was detected using HEt (right panel). Each histogram is representative of three experiments. Compared to NP69 cells, NP69-LMP1 cells exhibit significantly higher level of ROS contents, including hydrogen peroxide and nitrogen oxide (p<0.05).

Most cancer cells are under oxidative stress, which is associated with increased metabolic activity and ROS generation. LMP1 is the major transforming protein of EBV and behaves as a classical oncogene in rodent fibroblast transformation assays [12]. In the NP69 immortalized nasopharyngeal epithelial cell line, LMP1 expression induced a morphological change from a typical epithelial cobblestone shape to an elongated and fibroblastoid shape (S1A and S1B Fig). In serum-free keratinocyte medium supplemented with growth factors, NP69-LMP1 cells proliferated rapidly with an average doubling time of 34 h. In contrast, the NP69 cell proliferation rate was considerably reduced with a doubling time of 70 h (i.e., 2-fold longer than NP69-LMP1 cells, S1C Fig). Importantly, LMP1-transformed NP69 cells exhibited significantly increased basal ROS levels (approximately 8- and 10-fold increased) as quantified by flow cytometry using CM-H_2_DCF-DA and DCF-FM fluorescent probes, which are specific for hydrogen peroxide and nitrogen oxide, respectively (Fig 1A and 1E, fluorescence presented using a logarithmic scale). These data suggested that LMP1 expression causes cellular ROS accumulation in nasopharyngeal epithelial cells.

Next, we compared basal hydrogen peroxide level between NPC cells (C666-1, CNE-2 and SUNE-1) and immortalized nasopharyngeal epithelial cells (NP69 and NP69-LMP1). NPC cells exhibited a considerably reduced level of ROS compared with immortalized nasopharyngeal epithelial cells (Fig 1A, fluorescence presented using a logarithmic scale). Cellular ROS levels are a reflection of ROS generation as well as ROS elimination. The glutathione (GSH) antioxidant system and multiple enzymes, including catalase, superoxide dismutase (SOD) and GSH peroxidase (GPX), are expressed in cells to remove excessive ROS. Emerging evidence suggests that antioxidant systems are activated in cancer cells to suppress excessive ROS accumulation [13]. By immunoblotting, we demonstrate that the lower levels of ROS observed in NPC cells potentially resulted from increased levels of the antioxidant enzymes SOD2 (C666-1) and catalase (CNE-2 and SUNE-1, Fig 1B). Increased GSH levels in NPC cells might also be responsible for the observed reduction in ROS levels (Fig 1C). However, compared to NP69 cells, no significant difference in the expression of antioxidant enzymes or GSH levels were observed in LMP1-overexpressing NP69 cells (Fig 1D and 1E). This result implies that the increased ROS levels observed in NP69-LMP1 cells were due to increased ROS generation rather than decreased ROS elimination.

### LMP1-activated NOX and its role in ROS accumulation in NP69-LMP1 cells

**Fig 2:**
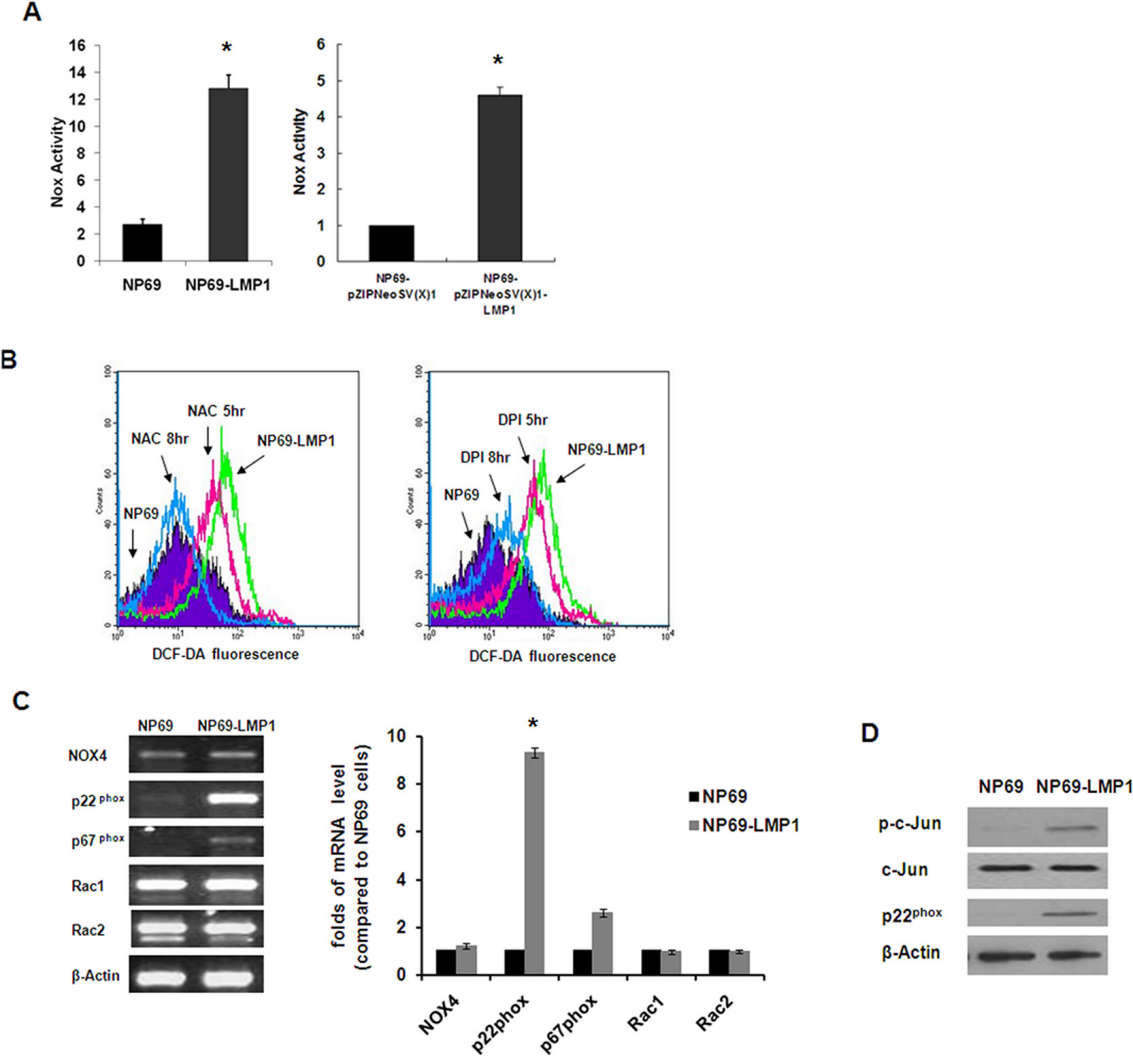
Oncogenic transformation by LMP1 causes increased NOX activity. **A:** Comparison of NOX activity in NP69 and NP69-LMP1 cells (left panel), NP69-pZIPNeoSV(X)1 and NP69-pZIPNeoSV(X)1-LMP1 transient-transfected cells (right panel) measured by a luminometer using lucigenin in the presence of NADPH (mean ± SD of three experiments; * p < 0.01). **B:** In NP69-LMP1 cells, ROS level was suppressed by both 0.5 mM NAC (34% at 5 hr and 83% at 8 hr, left panel) and 5 μM DPI (22% at 5 hr and 78% at 8 hr, right panel). ROS level was measured by flow cytometry using DCF-DA. Each histogram is representative of three experiments. **C:** Expression profile of NOX family subunits by RT-PCR in NP69 and NP69-LMP1 cells. LMP1 induced a significant increase in p22^phox^ expression. β-Actin served as a loading control. Compared to NP69 cells, p22^phox^ expression was significantly higher in NP69-LMP1 cells (*p<0.001). **D:** Basal protein expression of c-Jun, phosphorylated-c-Jun and p22^phox^ in NP69 and NP69-LMP1 cells. β-Actin served as a loading control.

Next, to avoid the interference from the antioxidant system in NPC cells, we used NP69 and NP69-LMP1 cells to extensively investigate the potential molecular mechanism involved in LMP1-mediated ROS generation. By consuming NAD(P)H and transferring electrons across biological membranes, the NOX family is an important intracellular source of superoxide and downstream ROS [14]. As shown in Fig 2A, compared with parental NP69 cells, LMP1-transformed NP69 cells exhibited a significant increase (nearly 5-fold) in NOX activity, as quantified by lucigenin chemiluminescence in the presence of NAD(P)H using a luminometer. Then, we used a pZIPNeoSV(X)1-LMP1 transient transfection system. As shown in Fig 2A, compared with NP69 cells transfected with empty vector, pZIPNeoSV(X)1-LMP1 transfection also significantly increased NOX activity (approximately 4.6 folds; p < 0.001). To further characterize the effect of NOX on intracellular ROS levels, we treated NP69-LMP1 cells with DPI and N-acetylcysteine (NAC). NAC is an effective antioxidant compound that boosts cellular GSH. Using DCF-DA and flow cytometry, we revealed that LMP1-induced ROS accumulation was reduced by NAC (34% and 83% decrease at 5 and 8 hr, respectively). However, LMP1-induced ROS accumulation was only partly rescued by DPI (22% and 78% decrease at 5 and 8 hr, respectively; Fig 2B). These observations suggest that the observed LMP1-induced ROS accumulation was not exclusively caused by activation of NOX family members. Nevertheless, to further identify which NOX family member plays a vital role in nasopharyngeal epithelial cells, we screened NOX subunit expression profiles in NP69 and NP69-LMP1 cells using RT-PCR. As shown in Fig 2C, among the five catalytic enzymes assessed (NOX1–5), only NOX4 mRNA was expressed at a detectable level in both NP69 and NP69-LMP1 cells. More importantly, mRNA of the regulatory subunit p22^phox^ was significantly upregulated in LMP1-transformed cells compared with parental NP69 cells. Immunoblotting data further confirmed p22^phox^ upregulation in LMP1-overexpressing NP69 cells (Fig 2D). Shiose et al. demonstrated that NOX4 consistently colocalizes with p22^phox^ at the cellular membranes and requires p22^phox^ for its catalytic activity [15]. Together, these data suggest that NOX4 and upregulated p22^phox^ may represent effective targets for the NOX inhibitor DPI in nasopharyngeal epithelial cells.

### LMP1 upregulates NOX subunit p22phox by activating the c-Jun N-terminal kinase(JNK) pathway

**Fig 3:**
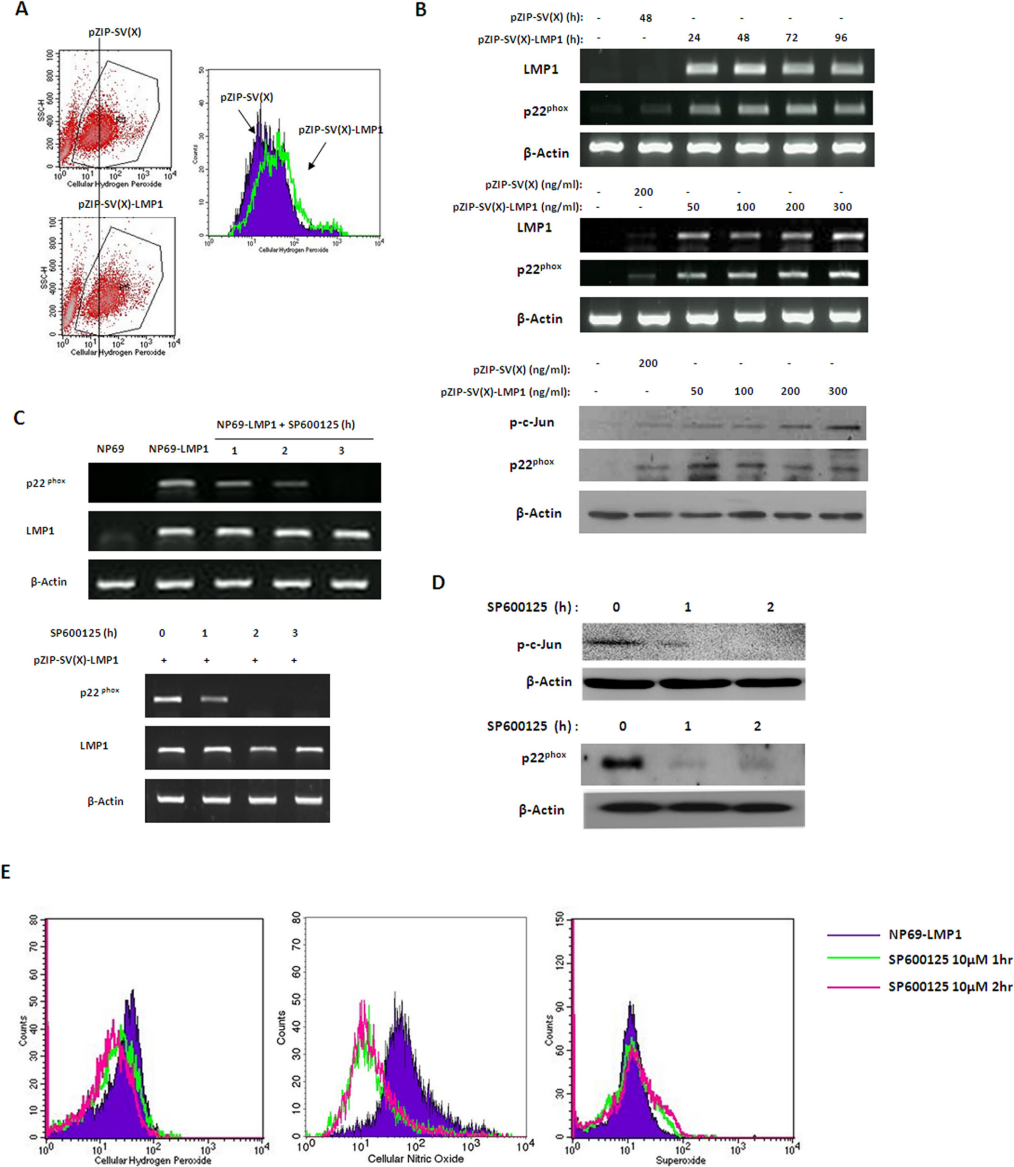
Inhibition of the JNK pathway downregulated the expression of p22^phox^. **A:** NP69 cells were transiently transfected with 200 ng/mL empty pZIP-SV(X) plasmid or pZIP-SV(X)-LMP1 plasmid for 48 hr. The increase in ROS in NP69-LMP1 cells was detected by flow cytometry using DCF-DA. Because transient transfection could cause some cells to die and this group of cells could not be stained with DCF-DA, we gated the healthy population to avoid the invalid signal. Each histogram is representative of three experiments. **B:** NP69 cells were transfected with 200 ng/mL empty pZIP-SV(X) plasmid or 50 to 400 ng/mL pZIP-SV(X)-LMP1 plasmid for 48 hr (upper panel), and NP69 cells were then transfected with 200 ng/mL pZIP-SV(X)-LMP1 plasmid for 24 to 96 hr (middle panel). The expression levels of LMP1 and p22^phox^ in NP69 cells transfected with LMP1 were measured by RT-PCR. NP69 cells were transfected with 200 ng/mL empty pZIP-SV(X) plasmid or 50 to 300 ng/mL pZIP-SV(X)-LMP1 plasmid for 48 hr (lower panel). The phosphorylation of c-Jun and the upregulation of p22^phox^ in LMP1-transfected NP69 cells were detected by an immunoblotting assay. β-Actin served as a loading control. **C:** Effects of JNK inhibitor SP600125 on the transcription of p22^phox^ and LMP1 in NP69-LMP1 cells (upper panel) and NP69 cells transiently transfected with 200 ng/mL pZIP-SV(X)-LMP1 plasmid for 48 hr (lower panel). β-Actin served as a loading control. **D:** Effects of SP600125 on the protein expression level of p22^phox^ and phosphorylation of c-Jun in NP69-LMP1 cells. NP69-LMP1 cells were incubated with 10 μM SP600125 for 1 or 2 hr, and cell lysates were analyzed by immunoblotting. β-Actin served as a loading control. **E:** Effect of SP600125 on the cellular ROS level in NP69-LMP1 cells. Each histogram is representative of three experiments.

LMP1 functions as a constitutively active tumor necrosis factor receptor (TNFR) and activates a number of signaling pathways, including NF-κB, JNK and STAT. In addition, researchers have recently demonstrated that the JNK pathway is responsible for the specific expression of the NOX regulatory subunit p22^phox^ [16]. Therefore, we hypothesized that LMP1 upregulates the NOX subunit p22^phox^ by activating the JNK pathway. Using TRANSFAC software, we identified a putative AP-1 binding site in the p22^phox^ promoter region. In addition, immunoblotting data indicated that both c-Jun phosphorylation levels and p22^phox^ expression levels were significantly upregulated in NP69-LMP1 cells compared with NP69 cells (Fig 2D). To investigate the molecular mechanism involved in the ellular redox status changes, we used a pZIPNeoSV(X)1-LMP1 transient transfection system. As presented in Fig 3A, compared with NP69 cells transfected with an empty vector, pZIPNeoSV(X)1-LMP1 transfection altered the cellular ROS level, promoting an obvious increase (greater than 2-fold) in cellular hydrogen peroxide concentrations, as quantified by flow cytometry using the DCF-DA fluorescent probe. Furthermore, p22^phox^ mRNA and LMP1 expression were significantly increased (Fig 3B). In addition, along with LMP1 expression, c-Jun phosphorylation and p22^phox^ protein expression were also detected by immunoblotting analysis in NP69 cells transfected with pZIPNeoSV(X)1-LMP1 (Fig 3B). These findings suggested that the JNK pathway was activated by LMP1 and this pathway potentially mediates LMP-induced p22^phox^ upregulation.

To further assess the role of activated JNK in LMP1-mediated p22^phox^ upregulation and ROS accumulation, we treat LMP1-transformed nasopharyngeal epithelial cells with the JNK inhibitor SP600125. As shown in Fig 3C, p22^phox^ mRNA expression in NP69-LMP1 cells was significantly inhibited by 10 μM SP600125 at 1 hr and completely suppressed at 3 h. Immunoblotting demonstrated that SP600125 also inhibited p22^phox^ expression at the protein level with 2 h in NP69-LMP1 cells (Fig 3D). Given the crucial role of p22^phox^ in ROS generation that that the SP600125-mediated JNK pathway inhibition completely inhibited p22^phox^ activation, SP600125 treatment should also cause cellular ROS depletion. Indeed, flow cytometry analysis revealed that 10 μM SP600125 caused significant decreases in cellular hydrogen peroxide (47%) and nitrogen oxide (21%) levels in NP69-LMP1 cells (Fig 3E). These data imply that the JNK pathway activated NOX subunit p22^phox^ at the transcriptional level, which subsequently affected the redox status in LMP1-expressing cells.

### p22phox expression levels are correlated with LMP1 expression levels in patients

**Fig 4:**
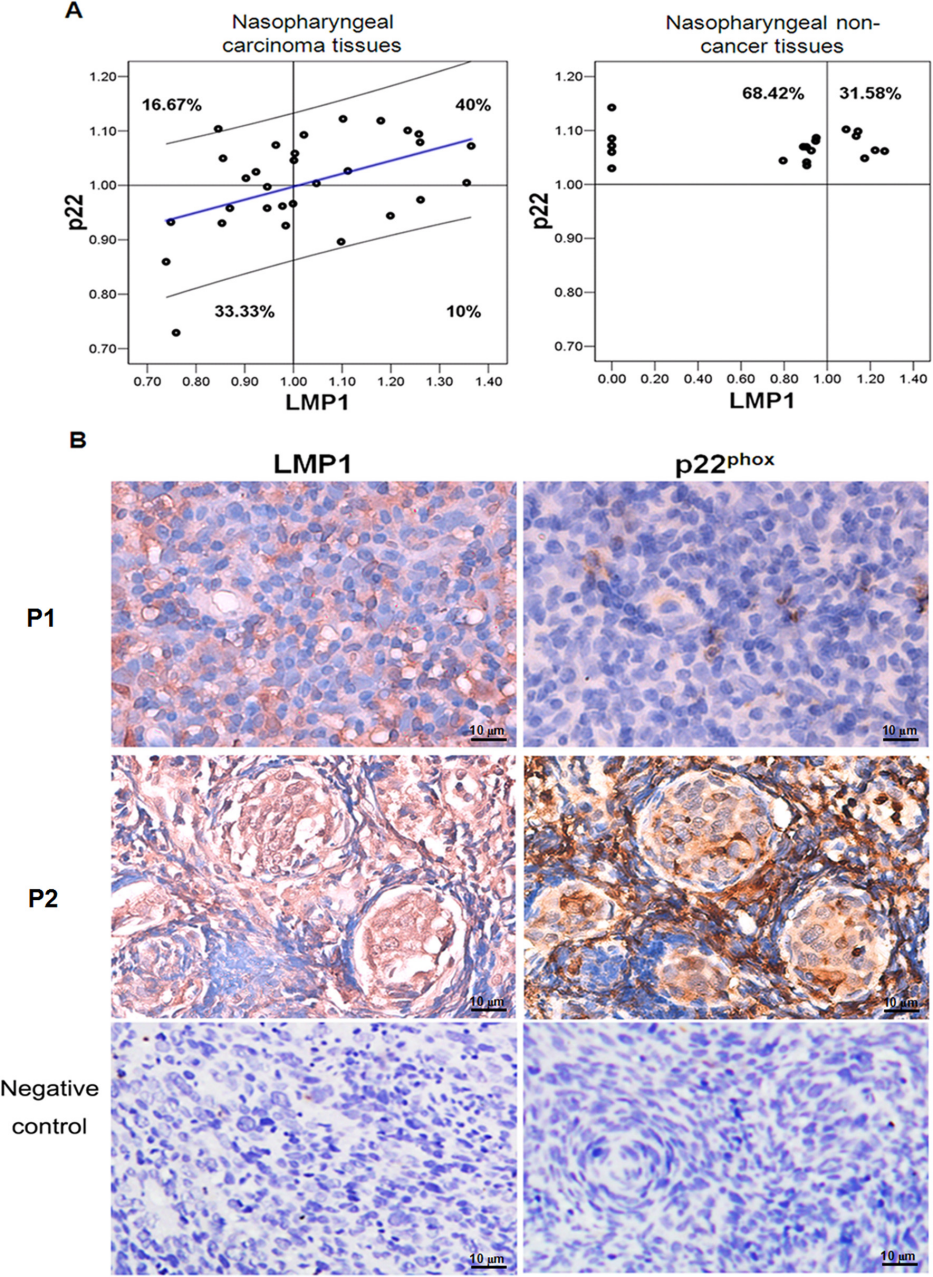
A positive correlation between LMP1 and p22^phox^ expression was detected in nasopharyngeal carcinoma. **A:** The distributions of NPC tissue samples and non-NPC tissue samples with LMP1^high^/ p22^phox high^, LMP1^low^/p22^phox low^, LMP1^high^/p22^phox low^ and LMP1^low^/p22^phox high^ were determined with SPSS correlation analysis. **B:** In a tissue array, the p22^phox^ and LMP1 expression levels were detected in NPC cancer tissues using an IHC assay. P1 and P2 represents two NPC tissues from patients with distinctive p22^phox^ and LMP1 expression pattern. P1 tissue has low p22^phox^ and LMP1 expression level, and P2 has higher LMP1 and p22^phox^ expression level. NPC tissue sample with LMP1 negative and p22^phox^ were served as negative control (magnification × 400).

Given the role of p22^phox^ in LMP1-mediated NOX activation and the metabolic disorder characterizing NPC cells, we investigated whether p22^phox^ overexpression is associated with LMP1 expression in NPC patients. We examined p22^phox^ and LMP1 expression levels in 30 NPC and 19 non-cancerous tissue samples. In NPC tissue samples, p22^phox^ expression was significantly correlated with LMP1expression (n = 30, p = 0.007, Table 1), whereas no significant correlation between p22^phox^ and LMP1 mRNA levels was detected in nasopharyngeal non-cancerous tissues (n = 19, p = 0.679). Approximately 40% of NPC samples were LMP1^high^/ p22^phox high^, and 33.33% of the NPC samples were LMP1^low^/p22^phox low^. In contrast, the percentages of samples with LMP1^high^/p22^phox low^ and LMP1^low^/p22^phox high^ correlations were lower (10% and 16.67%, respectively). However, out of the 19 non-cancerous tissues, LMP1 expression was only detected in 14 samples, whereas p22^phox^ was equally detected in both the LMP1^negative^ and the LMP1^high^ samples (Fig 4A). Of the 30 patients whose tumor biopsies were used in the NPC tissue assay (Fig 4B), p22^phox^ levels were also correlated with LMP1 expression in NPC tissues. P1 and P2 represents the NPC tissues from patients P1 and P2 with distinctive p22^phox^ and LMP1 expression pattern. P1 tissue has low p22^phox^ and LMP1 expression level, and P2 has higher LMP1 and p22^phox^ expression level. NPC tissue sample with LMP1 negative and p22^phox^ were served as negative control. These data confirmed the correlation between p22^phox^ and LMP1 expression in NPC tissue samples.

**Table 1:** Analysis of the correlation between LMP1 and p22^phox^ expression NPC tissues and non-cancer tissues.

	No. of Samples	Pearson Correlation	Significance(2-tail)
**Cancer**	**30**	**0.48**	**0.007**
**Non-Cancer**	**19**	**0.102**	**0.679**

**Note:** In total, 30 NPC tissues from patients and 19 non-cancer tissues were collected and analyzed by real-time PCR. The correlation was calculated using Pearson’s method. In NPC tissue samples, p22^phox^ mRNA expression positively correlated with LMP1 mRNA (p = 0.007). In non-cancer tissues, p22^phox^ mRNA expression did not correlate with LMP1 expression (p = 0.27).

### Activation of the glycolytic pathway in NP69-LMP1 cells

**Fig 5:**
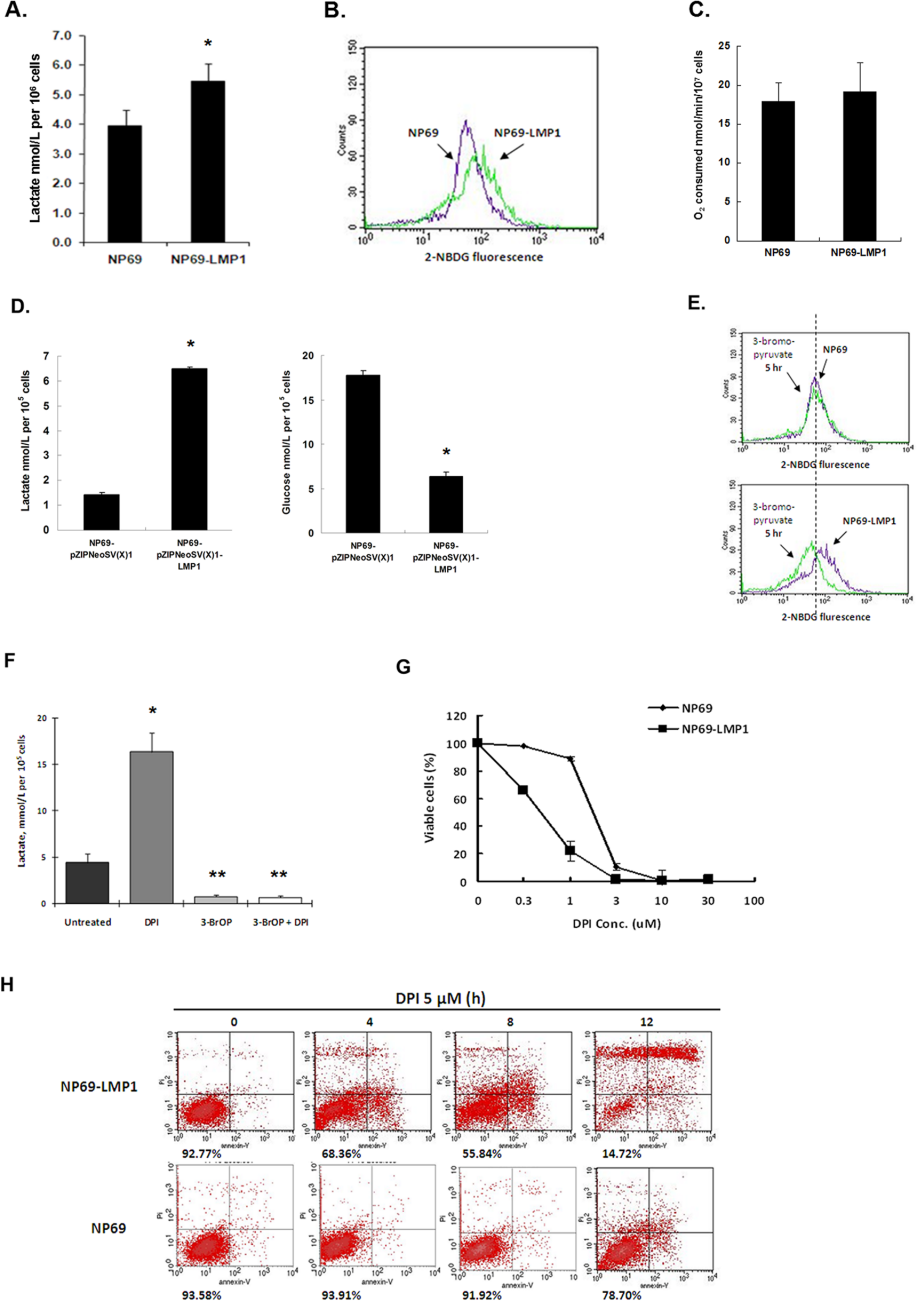
LMP1 causes a high glycolytic level and induces vulnerability to DPI. **A:** Lactate production in NP69 and NP69-LMP1 cells. Cells were incubated in KSF medium at a density of 5 × 10^5^ cells/mL for 24 hr. The lactate concentration in the medium was measured using an Accutrend Lactate Analyzer as described in Materials and Methods (mean ± SD of three experiments, * p<0.05). **B:** Comparison of glucose uptake in NP69 and NP69-LMP1 cells for 1 hr. Cells were incubated in glucose-free RPMI 1640 for 2 hr. Glucose uptake was detected by flow cytometry using a fluorescent deoxyglucose analog (2-NBDG). Each histogram is representative of three experiments. **C:** Oxygen consumption in NP69 and NP69-LMP1 cells (mean ± SD of three experiments, p = 0.5106). **D: Comparison of** lactate generation (left panel) and glucose consumption (right panel) in NP69-pZIPNeoSV(X)1 and NP69-pZIPNeoSV(X)1-LMP1 transient-transfected cells. Medium lactate and glucose concentration (nmol/L per 10^5^ cells, mean ± SD of three experiments, * p<0.05) were measured as described in Materials and Methods. **E:** An inhibitor of glucose uptake, 3-bromopyruvate, was used to test the glucose uptake in NP69 and NP69-LMP1 cells. Each histogram is representative of three experiments. **F:** Effect of combining DPI and 3-bromopyruvate on lactate production in NP69-LMP1 cells. A volume of 30 μM 3-bromopyruvate completely inhibited lactate production in NP69-LMP1 cells. When combined with 3-bromopyruvate, 5 μM DPI treatment could not induce any increase in lactate (mean ± SD of three experiments). * p<0.05, DPI treated group vs untreated control group; ** p<0.05, 3-BrOP treated group alone or combined with DPI vs DPI treated group. **G:** Treatment with 0.3–30 μM DPI preferentially killed NP69-LMP1 cells compared to NP69 cells (mean ± SD of three experiments). **H:** Preferential killing of NP69-LMP1 cells by 5 μM DPI was detected by annexin V-PI staining and flow cytometry. The numbers under the plots indicate live cells with both low annexin V staining and low PI staining. Each histogram is representative of three experiments.

Under hypoxia and oxidative stress, cancer cells are more dependent on anaerobic respiration and the glycolytic pathway to meet excessive bioenergetics needs [17]. To evaluate the influence of LMP1-induced oxidative stress on energy metabolism, we examined the glycolytic activity in NP69 and NP69-LMP1 cells. Glycolytic activity can be evaluated using the glycolytic index as calculated using the following formula: (lactate generation rate × glucose uptake rate) / oxygen consumption rate [9]. As shown in Fig 5A and 5B, compared with NP69 cells, NP69-LMP1 cells produced more lactate (1.4-fold increase, p = 0.02) and exhibited a increased basal level of glucose uptake (1.5-fold) as quantified by flow cytometry using 2-NBDG. However, compared with NP69 cells, NP69-LMP1 cells did not exhibit an obvious decrease in oxygen consumption (Fig 5C) Increased glucose uptake and lactate accumulation indicate increased glycolytic activity in NP69-LMP1 cells, with a greater than 2-fold increased glycolytic index compared with NP69 cells. Additionally, using a transient transfection system (Fig 5D), we found that, pZIPNeoSV(X)1-LMP1 transfection significantly increased both lactate generation (approximately 4.57-folds increase) and glucose consumption as demonstrated by an approximately 65% reduction in glucose the medium compared with NP69 cells transfected with empty vector (p < 0.001). The PI3K/Akt pathway directs cancer cells towards aerobic glycolysis by activating the c-Myc and mTOR pathways [18,19]. Furthermore, LMP1 activates PI3K/Akt signaling in LMP1-mediated transformation [20], which suggests that LMP1 might activate glycolysis via the Akt pathway.

### DPI treatment promotes lactate generation in nasopharyngeal epithelium cells

High glycolysis rates produce significant amounts of lactate. The glycolysis inhibitor 3-BrOP, an optimized derivative of 3-bromopyruvate (3-BrAP), is an inhibitor of hexokinase [21]. Upon treatment with the glycolysis inhibitor 3-BrOP for 5 h, NP69-LMP1 cells exhibited a significant decrease (approximately 50%) in 2-NBDG fluorescence levels, whereas no obvious response was detected in NP69 cells at the same time point (Fig 5E). Treatment with 3-BrOP also inhibited lactate generation in NP69-LMP1 cells. Interestingly, the NOX inhibitor DPI significantly increased (3.3-fold) lactate in NP69-LMP1 cells (Fig 5F), which might imply a role of NOX in controlling lactate generation. As shown in Fig 5F, DPI treatment did not reverse the inhibitory effect of 3-BrOP. This finding implies that the glycolytic enzyme targeted by DPI acts downstream within the glycolytic pathway. In this study, we focused on the biological effect of NOX and its inhibitor DPI but did not comprehensively investigate all the potential effects of NOX on glycolysis. In our future studies, we will assess the detailed molecular mechanism involved in NOX-mediated glycolysis regulation in nasopharyngeal carcinoma.

### Cytotoxic effect of DPI in cells with high levels of Nox activity and p22phox expression

Given that p22^phox^ is highly expressed in NP69-LMP1 cells, we hypothezised that the NOX inhibitor DPI could target over-expressed p22^phox^ and cause cell death. As shown in Fig 5G, compared with NP69 cells, DPI treatment preferentially killed NP69-LMP1 cells in a dose-dependent manner after 72 h. NP69-LMP1 cells were more vulnerable to DPI. Moreover, annexin V-PI staining and flow cytometry analysis demonstrated that 5 μM of DPI effectively induced cell death in NP69-LMP1 cells in a time-dependent manner, causing apoptosis in approximately 31%, 44% and 85% of cells at 4, 8 and 12 h, respectively. In contrast, NP69 cells were significantly less sensitive to 5 μM DPI at all time points assessed, with only 6.09%, 8.08% and 21.30% of cells dying at 4, 8 and 12 h, respectively (Fig 5H).

A latent EBV infection is crucial in NPC tumorigenesis, and LMP1 protein is detected in 68% of NPC patients by immunoblotting [22]. Despite the antioxidant systems suppressing ROS in NPC cells, LMP1 stimulates oxidative stress in these cells. Evidence for this effect is presented in S2A and S2B Fig Indeed, in CNE-1 cells, LMP1 expression greatly elevated glutathione disulfide (GSSG) levels, reduced the GSH/GSSG ratio, and upregulated the expression of the redox-sensitive factor nuclear respiratory factor 1 (NRF1). Additionally, LMP1 enhanced lactate generation in CNE1 cells (S2C Fig).

**Fig 6:**
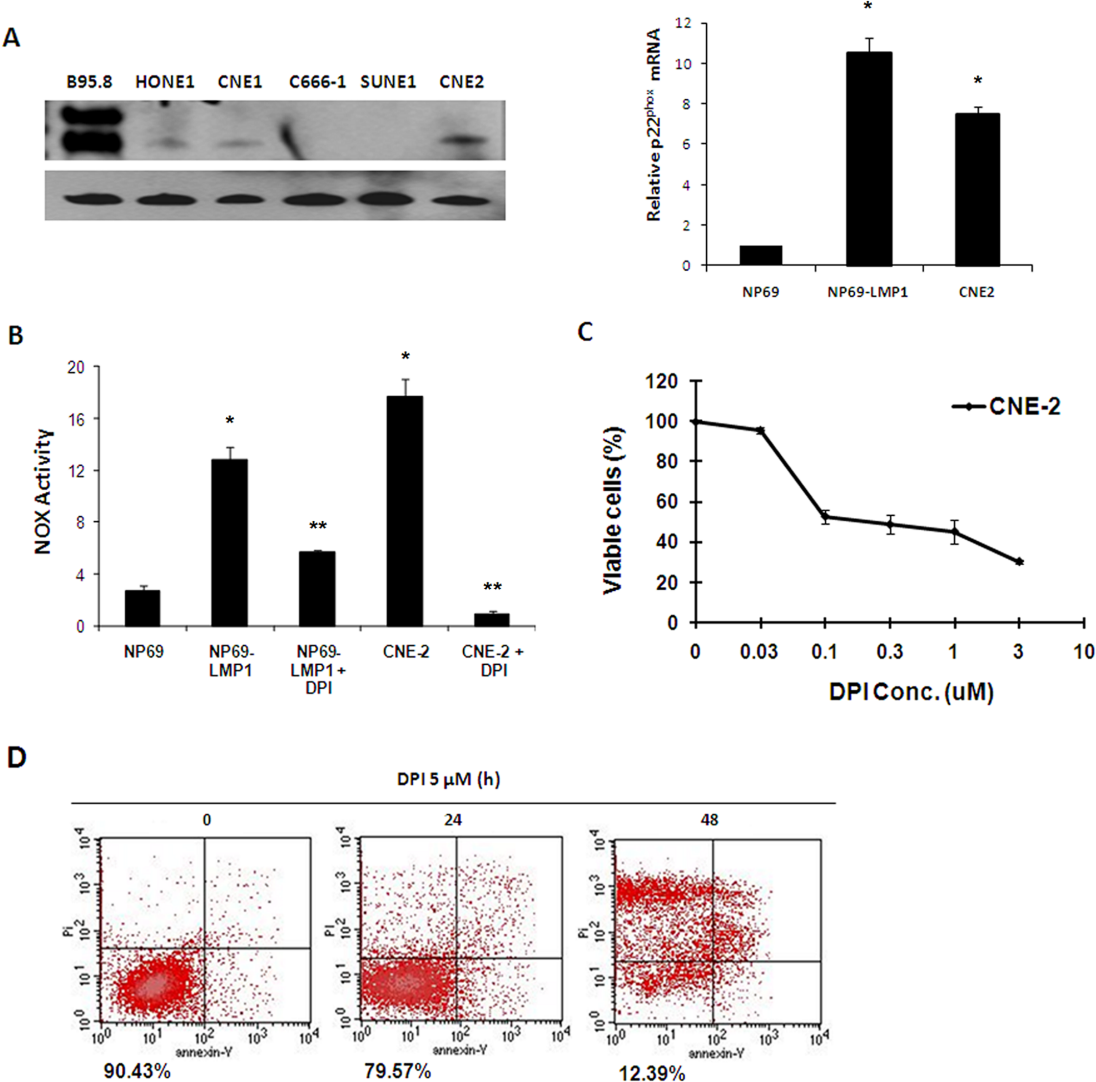
NOX activation makes NPC cells vulnerable to the NOX inhibitor DPI. **A:** p22^phox^ mRNA expression level was evaluated in NP69, NP69-LMP1 and CNE2 cells by RT-PCR and real-time quantitative PCR. LMP1 expression was detected in NPC cells and B95.8 B lymphoma cells. β-Actin served as a loading control (mean ± SD of three experiments). Compared to NP69 cells, NP69-LMP1 and CNE2 cells had significantly higher p22^phox^ mRNA expression level (*p<0.01). **B:** Comparison of NOX activity and the effect of DPI on NOX activity in NP69, NP69-LMP1 and CNE2 cells, measured by a luminometer using lucigenin in the presence of NADPH (mean ± SD of three experiments). Compared to NP69 cells, NP69-LMP1 and CNE2 cells had significantly higher NOX activity (* p < 0.01). DPI treatment could significantly suppress NOX activity in NP69-LMP1 and CNE2 cells (** p<0.01). **C:** Mortality effect of 0.03–10 μM DPI on CNE2 NPC cells, evaluated using an MTT assay (mean ± SD of three experiments). DPI suppressed CNE2 cells proliferation. **D:** Mortality effect of 5 μM DPI on CNE2 cells, detected using annexin V-PI staining and flow cytometry. The numbers under the plots indicate the live cells with both low annexin V staining and low PI staining. Each histogram is representative of three experiments.

In NPC cells (CNE2 cells, Fig 6A and 6B), NOX was also activated and p22^phox^ expression was upregulated. These data suggest that NOX activation might also play an important role in malignant transformation and metabolic disorders in NPC cells that lack LMP1 expression. Next, we assessed the effects of DPI on CNE2 cells. Based on MTT assay, DPI treatment (0.1–3 μM) significantly inhibited proliferation in CNE2 cells at 72 h (Fig 6C). Annexin V-PI staining and flow cytometry analysis further confirmed the effect of DPI treatmen t(5 μM) in CNE2 cells, with approximately 90% apoptotic cells at 24 hr (Fig 6D). These findings suggested that LMP1-stimulated NOX activation plays important roles in EBV-related malignant cells. Thus, DPI-mediated inhibition of NOX activity could effectively induce cell death in these cancer cells.

## Discussion

Oncoviruses, such as EBV, hepatitis B virus, HTLV-I and some human papillomaviruses, contribute to the development of a large proportion of human tumors. Experimental data indicate a clear role for some of the HPV-encoded latent genes in tumor cell growth [23]. Similar to HPV, EBV is a common infectious virus that persists after primary infection in a latent state with occasional viral shedding. EBV infections occur worldwide. In the US, up to 95% of adults between the age of 35 and 40 years old have been infected. Although EBV infection is benign in the majority of humans, various types of human tumors are associated with EBV infection, including Burkitt’s lymphoma, Hodgkin lymphoma, NPC, gastric carcinoma, breast cancer, hepatocellular carcinoma and follicular dendritic cell sarcoma. Why does EBV, which infects humans in their youth, evolve to initiate tumor formation later in life? According to K.W. Lo and D.P. Huang (personal communication), the proposed NPC tumorigenesis model stipulates that EBV latently infects nasopharyngeal epithelial cells during the stage of low-grade dysplasia and require a long time to develop into invasive carcinoma. In addition, latent viral proteins play important roles in NPC tumorigenesis [24].

Oxidative stress refers to a cellular state characterized by excessive production of ROS and/or reduced antioxidant defenses responsible for ROS metabolis. Moreover, oxidative stress is involved in the pathophysiology of numerous human diseases, including cancer. ROS have been implicated at all stages of the carcinogenic process, including proliferation, senescence, inflammation and metastasis, via both genotoxic mechanisms (mutations) and non-genotoxic mechanisms indirectly affecting DNA to modulate gene expression [25]. In this study, we found that LMP1 induces excessive ROS generation by upregulating the expression of p22^phox^ and NOX activity (Fig 2) through the JNK/AP-1 signaling pathway in nasopharyngeal epithelial cells without affecting the antioxidant system (Fig 1). Previous studies have reported that ROS are downstream products of TNF-R-associated factor (TRAF)-mediated signal transduction [26] and that several members of the NOX family, including NOX1, NOX4, p47^phox^ and p22^phox^, are regulated by JNK/AP-1 [16]. Based on these clues, our study first identified the signaling pathway responsible for the LMP1-induced ROS generation and suggested that excessive ROS production by NOX facilitates LMP1-medicated malignant transformation of human nasopharyngeal epithelial cells.

Oxygen metabolism disorders and cellular proliferation engage opposite cellular pathways that often coexist during tumor growth. To sustain cellular bioenergetics and malignant growth, both hypoxia and a key transcription factor, c-Myc, promote tumor glycolysis by acting on pivotal glycolytic pathway enzymes, such as glucose transporter 1, hexokinase II, lactate dehydrogenase(LDH) and pyruvate dehydrogenase kinase 1 [27,28]. In our model, activated NOX consumes cellular oxygen and causes a certain degree of hypoxia; whereas, the LMP1-activated PI3K-Akt pathway stimulates glycolysis.

At a high glycolytic rate, cancer cells overproduce the final product of anaerobic glycolysis, lactate, and tolerate this hyperacid cellular environment, whereas normal cells suffer. NP69 and NP69-LMP1 cells, which were used in our study to mimic the early stage of NPC tumorigenesis, are immortalized normal epithelial cells and pre-malignant transformed epithelial cells, respectively. Neither cell line could tolerate the hyperacid environment. Therefore, to avoid lactate overproduction in the early stage of tumor formation, LMP1-transformed cells must consume as much cellular free NAD(P)H as possible to prevent LDH from using this substrate to produce excessive lactate. Our data indicate that activated NOX consumes the free cellular NAD(P)H in LMP1-transformed NP69 cells. Upon inhibtion of NOX activity using DPI, significant lactate amounts were produced, and NP69-LMP1 cells underwent apoptotic cell death. In this network, p22^phox^ plays a crucial role in both the generation of ROS and the maintenance of high glycolytic metabolism without acidosis. Therefore, our study is a landmark in studies involving the role of EBV latent infection in early metabolic changes during NPC tumorigenesis of NPC.

LMP1 can be detected in pre-invasive nasopharyngeal lesions and invasive carcinoma cases [29], suggesting that LMP1 expression is necessary for the transformation of early lesions. In addition, LMP1 may also play a malignancy-promoting role in invasive carcinoma. In addition, as shown in Fig 6 and S2 Fig, NOX was activated in malignant CNE2 and DPI induced their death. Out of 30 NPC tissues samples studied, some expressed LMP1 at a relatively high level, whereas other samples exhibited reduced LMP1 expression (Fig 4). p22^phox^ expression level were positively correlated with LMP1 expression in NPC samples, implying a correlation between NOX activation and LMP1 expression in NPC. We believe that DPI, a specific inhibitor of NOX, could be useful in suppressing tumor growth in LMP1^high^/ p22^phox high^ tumor cells in NPC patients.

## Conclusion

The oncovirus protein LMP1 stimulates ROS generation and glucose metabolism disorders in nasopharyngeal carcinoma cells via NOX activation. NOX inhibition with DPI might be useful to suppress tumor growth in LMP1^high^/ p22^phox high^ NPC patients.

## Supporting Information

S1 Fig Oncogenic transformation by LMP1 in immortalized nasopharyngeal epithelial cells. **A:** Expression of LMP1 in NP69 and NP69-LMP1 cells, measured by RT-PCR assays. **B:** Morphologies of NP69-immortalized nasopharyngeal epithelial cells. NP69 cells showed epithelial cobblestone morphology. NP69-LMP1 cells exhibited an elongated and fibroblast-like shape. All panels are of the same magnification; scale bar, 10 mm. **C:** Effect of LMP1 on the growth curve of NP69 cells. In medium supplemented with growth factors, NP69-LMP1 cells grow faster than NP69 cells (mean ± SD of three experiments).
(TIF)

S2 Fig LMP1 induces oxidative stress in NPC cells. **A:** Protein levels of LMP1 and NRF1 were detected by immunoblotting. β-Actin served as a loading control. **B:** Lactate production in CNE1 and CNE1-LMP1 cells. Cells were incubated in RPMI 1640 medium supplemented with 10% fetal bovine serum (FBS) at a density of 5 × 10^5^ cells/mL for 24 hr. The lactate concentration in the medium was measured using an Accutrend Lactate Analyzer as described in Materials and Methods (mean ± SD of three experiments; * p<0.01). CNE-LMP1 cells produced significantly higher level of lactate than CNE1 cells. **C:** Comparison of cellular GSH, GSSG, 5-oxoproline, cysteine and the GSH/GSSG ratio in CNE1 and CNE1-LMP1 cells (mean ± SD of three experiments).
(TIF)

S1 Table Summary of the primers used in the reverse transcriptase-PCR. **Note:** Primers corresponding to NOX subunits are listed in this table.
(TIF)
