# Supplementary figures and images for: LMP1 Increases Expression of NADPH Oxidase (NOX) and Its Regulatory Subunit p22 in NP69 Nasopharyngeal Cells and Makes Them Sensitive to a Treatment by a NOX Inhibitor
Source: PLoS One. 2015 Aug 5;10(8):e0134896. doi: 10.1371/journal.pone.0134896 (PMC4526464; doi:10.1371/journal.pone.0134896)

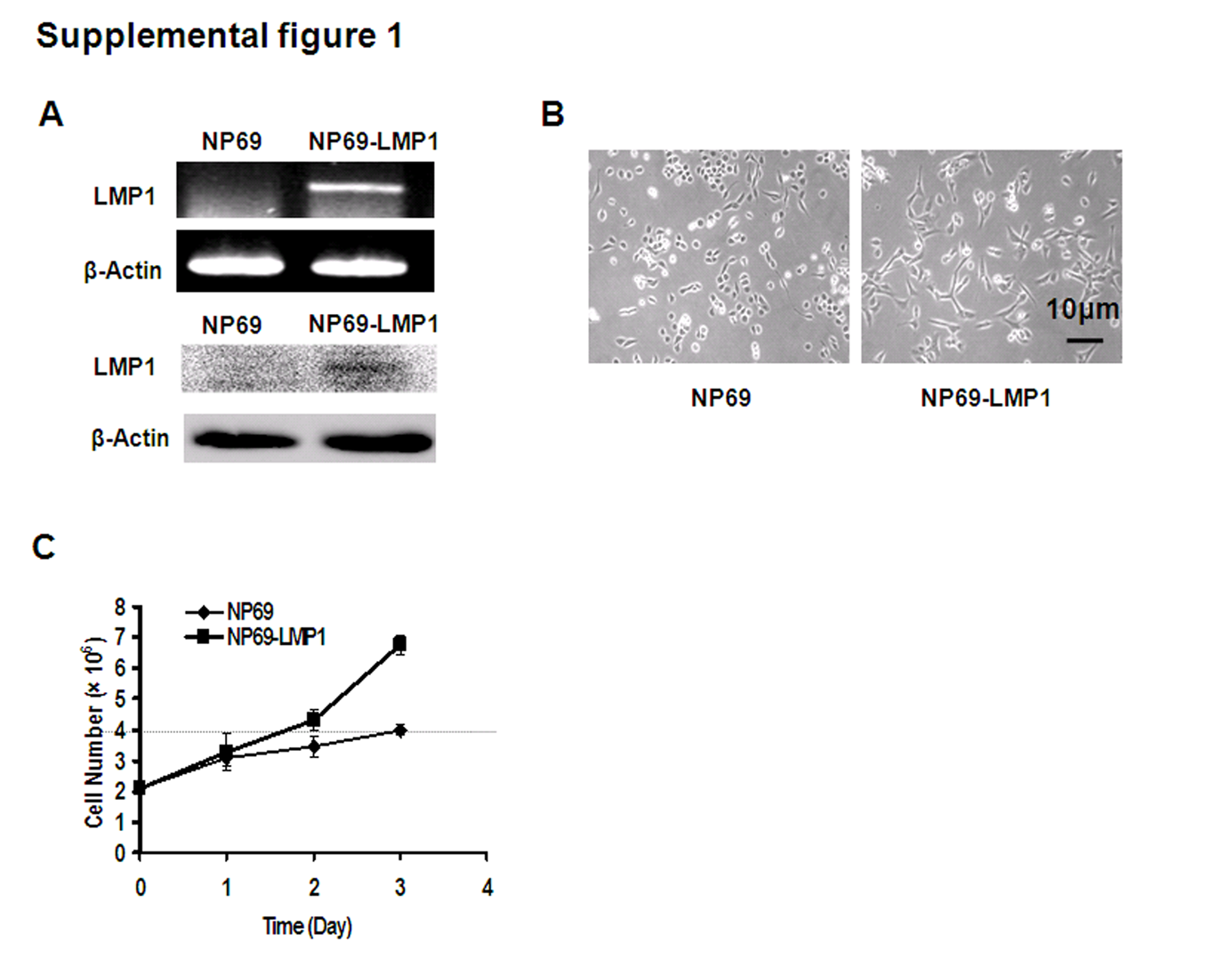

Supplement: S1 Fig — A: Expression of LMP1 in NP69 and NP69-LMP1 cells, measured by RT-PCR assays. B: Morphologies of NP69-immortalized nasopharyngeal epithelial cells. NP69 cells showed epithelial cobblestone morphology. NP69-LMP1 cells exhibited an elongated and fibroblast-like shape. All panels are of the same magnification; scale bar, 10 mm. C: Effect of LMP1 on the growth curve of NP69 cells. In medium supplemented with growth factors, NP69-LMP1 cells grow faster than NP69 cells (mean ± SD of three experiments). (TIF) [file pone.0134896.s001.tif]

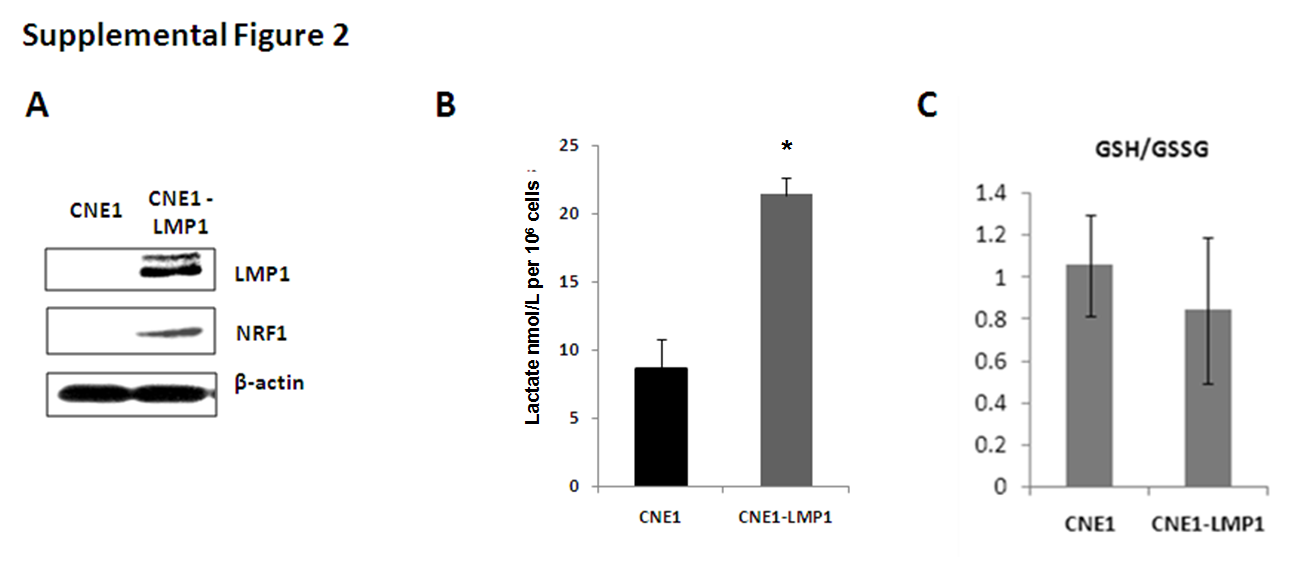

Supplement: S2 Fig — A: Protein levels of LMP1 and NRF1 were detected by immunoblotting. β-Actin served as a loading control. B: Lactate production in CNE1 and CNE1-LMP1 cells. Cells were incubated in RPMI 1640 medium supplemented with 10% fetal bovine serum (FBS) at a density of 5 × 10^5^ cells/mL for 24 hr. The lactate concentration in the medium was measured using an Accutrend Lactate Analyzer as described in Materials and Methods (mean ± SD of three experiments; * p<0.01). CNE-LMP1 cells produced significantly higher level of lactate than CNE1 cells. C: Comparison of cellular GSH, GSSG, 5-oxoproline, cysteine and the GSH/GSSG ratio in CNE1 and CNE1-LMP1 cells (mean ± SD of three experiments). (TIF) [file pone.0134896.s002.tif]

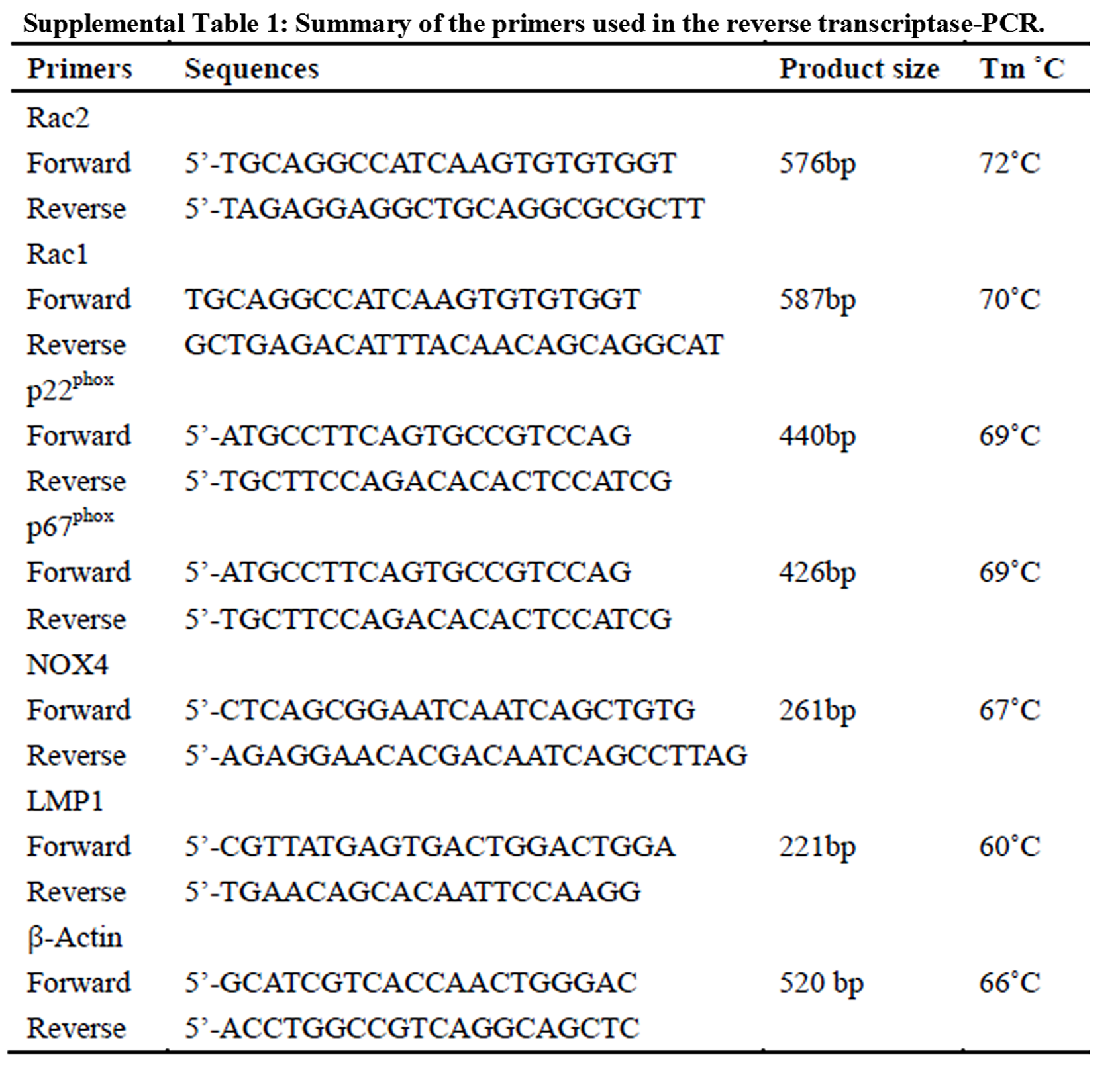

Supplement: S1 Table — Note: Primers corresponding to NOX subunits are listed in this table. (TIF) [file pone.0134896.s003.tif]
